# Beyond Sarcopenia: An Exploratory Machine Learning Analysis of Fatigue, Sleep Quality and Acute-Phase Severity as Correlates of Post-COVID Functional Status in a Colombian Cohort

**DOI:** 10.3390/biomedicines14091885

**Published:** 2026-08-24

**Authors:** Jorge Enrique Daza-Arana, Yamil Liscano, Rubén Eduardo Varela-Miranda, Heiler Lozada-Ramos, María Angélica Rodríguez-Scarpetta

**Affiliations:** 1 Physiotherapy Program, Faculty of Health, Universidad Santiago de Cali, Cali 5183000, Colombia; maria.rodriguez10@usc.edu.co; 2 Health and Movement Research Group, Faculty of Health, Universidad Santiago de Cali, Cali 5183000, Colombia; heiler.lozada00@usc.edu.co; 3 Comprehensive Health Research Group (GISI), Faculty of Health, Universidad Santiago de Cali, Cali 5183000, Colombia; 4 Research Group in Chemistry and Biotechnology (QUIBIO), Faculty of Basic Sciences, Universidad Santiago de Cali, Cali 5183000, Colombia; ruben.varela00@usc.edu.co

**Keywords:** post-COVID condition, functional status, fatigue, handgrip strength, machine learning, exploratory analysis, TRIPOD + AI, algorithmic fairness

## Abstract

**Background:** Handgrip strength has been proposed as an objective marker of post-COVID functional impairment, but its contribution relative to fatigue and sleep quality has not been formally evaluated in Latin American populations. **Methods:** We conducted an exploratory cross-sectional analysis of 130 adults with post-COVID condition in Palmira, Colombia. The outcome was any functional limitation on the Post-COVID Functional Status (PCFS) scale (grades 1–4 versus grade 0). Eight predictors were pre-specified on clinical grounds before examining outcome associations, and ridge-penalized logistic regression was pre-specified as the primary model. Sample size adequacy followed the criteria of Riley et al. Internal validation used bootstrap optimism correction (B = 1000), re-running the complete pipeline within each replicate. We assessed calibration, decision curves, prediction stability, SHapley Additive exPlanations (SHAP) with bootstrap rank intervals, and subgroup performance. Reporting followed TRIPOD + AI; risk of bias was self-assessed with PROBAST + AI. **Results:** Functional limitation affected 91/130 participants (70.0%). The minimum sample required for eight parameters was 566; the cohort met 23.0% of this requirement, so the analysis is exploratory. The optimism-corrected area under the curve was 0.748 (95% confidence interval (CI) 0.669–0.830), with calibration slope 1.15 (0.71–1.69). Fatigue ranked first in SHAP importance (54.0% of resamples), whereas grip strength ranked fifth (median rank 6). Net benefit exceeded both default strategies between thresholds 0.38 and 0.88. Thirty percent of participants changed classification in over 20% of resamples. **Conclusions:** Fatigue, sleep quality and acute-phase severity, rather than grip strength, emerged as the most influential correlates. These hypothesis-generating findings require confirmation in adequately powered cohorts.

## 1. Introduction

Post-COVID condition, characterized by symptoms persisting beyond twelve weeks after SARS-CoV-2 infection, affects a substantial proportion of people who have had acute coronavirus disease 2019 (COVID-19) and imposes a measurable burden on daily functioning [1,2]. The Post-COVID Functional Status (PCFS) scale, developed early in the pandemic, provides a brief ordinal measure of this burden and has been adopted widely because it captures gradations of limitation that binary outcomes such as survival or readmission cannot [3]. Cross-sectional work in Latin American settings has reported functional limitation in approximately 60% of adults assessed after acute COVID-19, with fatigue and impaired concentration among the most frequent persistent complaints [4,5]. The Colombian context adds specific considerations. Coinfection and superinfection were frequent during the acute phase in this country and may plausibly influence the severity of subsequent sequelae [6], and in Colombian intensive care cohorts, mortality was driven by clinical and ventilatory factors rather than by socioeconomic position or health insurance scheme [7], which suggests that the determinants of outcome in this health system are not reducible to social gradient alone. Colombian evidence is consistent with this pattern: a multicenter cross-sectional study of 1047 outpatients reported fatigue in 53.3% and sleep disorders in 35.7%, with 72% presenting four or more persistent symptoms [6], while a cohort study identified female sex, chronic obstructive pulmonary disease, rheumatic disease and mechanical ventilation during the index infection as factors associated with persistence at six months [8]. A comparative analysis of Colombian patients with neurological sequelae likewise found fatigue (74%) and sleep problems (46%) to be the most frequent non-neurological complaints [7].

Skeletal muscle involvement has attracted particular attention as a candidate mechanism. Reduced muscle strength and mass have been documented repeatedly after SARS-CoV-2 infection, and handgrip strength is the most accessible bedside proxy for global muscle function [9,10]. A Brazilian longitudinal study found that dynapenia, defined by low handgrip strength, accompanied worse pulmonary function and worse functional outcomes in long COVID [11]. Reduced limb strength in post-COVID syndrome appears to be mediated substantially by limb muscle mass rather than by an independent neuromuscular deficit [12], and a systematic review of musculoskeletal sequelae conducted by our group, appraising studies published between 2020 and 2025, confirmed that weakness, pain and sarcopenia recur across study designs while noting the heterogeneity of the instruments used to measure them [13]. These observations motivated the hypothesis that grip strength might serve as an objective, low-cost marker of functional recovery, particularly attractive in resource-constrained health systems where instrumented assessment is unavailable.

Two considerations temper that expectation. First, fatigue is consistently the single most prevalent post-COVID symptom and is itself strongly associated with worse PCFS grades; hierarchical models have attributed a large share of the variance in functional limitation to fatigue, dizziness and memory complaints combined [14,15]. Sleep disturbance and cognitive symptoms frequently co-occur with fatigue and may confound or mediate any apparent strength–function association [16]. Whether grip strength retains influence once fatigue and sleep quality are modeled jointly has not been formally tested. Second, muscle function in post-COVID syndrome is dynamic rather than static: multidimensional grip protocols capturing fatigability and recovery capacity distinguish patients from recovered controls better than peak force alone [17], which implies that a single maximal contraction may be an insensitive summary of the underlying pathology.

Machine learning (ML) has been applied extensively to COVID-19 prognosis, but the post-COVID literature is markedly less mature. Models predicting long COVID from acute-phase clinical and immunological data achieve median areas under the receiver operating characteristic curve (AUC) between 0.64 and 0.66 [18], and a large community challenge using data from more than 75 institutions concluded that interpretability and workflow feasibility, not headline discrimination, are the limiting factors for clinical adoption [19]. This modest performance ceiling is important context: it suggests that post-COVID functional trajectories are intrinsically difficult to predict and that studies reporting substantially higher discrimination in small samples warrant scrutiny for optimism.

Methodological standards for such studies have tightened. TRIPOD + AI provides a reporting framework specific to prediction models using regression or ML [20], and PROBAST + AI provides a structured risk-of-bias assessment [21]. Both emphasize items that small-sample studies commonly omit: a formal sample size justification, calibration alongside discrimination, and honest internal validation. The relevant sample size criteria of Riley et al. depend on the number of candidate parameters, outcome prevalence and anticipated discrimination and frequently reveal that published models were developed on cohorts an order of magnitude smaller than required [22,23]. A systematic review found that only 8% of prediction model studies for binary outcomes considered sample size at all and that 73% fell below the minimum [24]. Separately, simulation evidence now shows that oversampling corrections for class imbalance, including the synthetic minority oversampling technique, do not improve discrimination and severely miscalibrate predicted risks, with the harm increasing as sample size decreases [25,26].

Against this background, the present analysis had three objectives: first, to quantify how much of the between-person variation in post-COVID functional status is captured by a small, pre-specified set of clinical variables in a Colombian cohort; second, to establish the relative influence of grip strength against fatigue, sleep quality, cognition, comorbidity and acute-phase severity within the same model, rather than in isolation; and third, to characterize honestly the uncertainty attached to those estimates at the available sample size, including calibration, clinical utility and prediction stability. Given the sample size constraints quantified below, we frame this work as exploratory and hypothesis-generating and make no claim to a validated prediction tool.

## 2. Materials and Methods

### 2.1. Study Design and Population

We performed an exploratory cross-sectional analysis nested in a cohort of adults with post-COVID condition attending health services in Palmira, Valle del Cauca, Colombia. Eligible participants were adults aged 18 years or older with a documented history of SARS-CoV-2 infection confirmed by reverse transcription polymerase chain reaction or antigen testing and persistent symptoms twelve weeks or more after the acute episode. Participants unable to complete grip dynamometry because of upper-limb injury or unable to complete self-report instruments because of severe cognitive or sensory impairment were excluded. Of 139 records screened, 130 had complete outcome ascertainment and formed the analytic sample; the nine excluded records were empty rows with no outcome or predictor data. The twelve-week eligibility criterion served exclusively as a lower bound without an upper limit, as dates of infection, diagnosis, or assessment were not recorded in the dataset. Consequently, elapsed time since infection could not be quantified for any participant (see Section 4.6 for methodological implications).

This study was approved by the Scientific Ethics and Bioethics Committee of Universidad Santiago de Cali (Act No. 02/2025) and endorsed by the Palmira Municipal Health Secretariat. All participants provided written informed consent. Reporting follows the TRIPOD+AI statement, and a risk of bias self-assessment was conducted using PROBAST+AI (detailed in Supplementary Tables S6 and S7, respectively).

### 2.2. Outcome Variable

The outcome was functional limitation measured with the PCFS scale, which grades limitation from 0 (no limitation) to 4 (severe limitation) [3]. For the primary analysis, the scale was dichotomized into any limitation (grades 1–4) versus no limitation (grade 0). The full ordinal grade was retained for a pre-specified secondary analysis (Section 2.8). Dichotomization was chosen for the primary analysis because it corresponds to the referral decision the model is intended to inform, namely whether a patient should be assessed for rehabilitation; the ordinal analysis was added to test whether the dichotomy discarded useful information. The distribution across all individual PCFS grades (0 to 4) is provided in Supplementary Table S8 and aligns with the ordinal variable specified for secondary analyses.

### 2.3. Predictor Variables

Eight predictors were pre-specified on clinical grounds and fixed before any outcome association was examined: maximum handgrip strength, age, sex, total score on the Modified Fatigue Impact Scale (MFIS), global score on the Pittsburgh Sleep Quality Index (PSQI), total score on the Montreal Cognitive Assessment (MoCA), the Charlson comorbidity index, and hospitalization during the acute infection. The rationale for each is documented in a machine-readable configuration file distributed with the analysis code, so that the claim of pre-specification is verifiable rather than asserted.

The cap of eight parameters followed directly from the sample size assessment (Section 2.4). With 39 events in the smaller outcome class, eight parameters correspond to approximately five events per parameter, already at the limit of what is defensible. Persistent-symptom inventories comprising 40 individual items, socioeconomic stratum, insurance type, educational level and ethnicity were retained for descriptive and fairness analyses but deliberately excluded from the model. Socioeconomic stratum refers to the Colombian administrative classification of residential dwellings from 1 (lowest) to 6 (highest), used nationally to allocate subsidies and widely employed as a proxy for household socioeconomic position. Health insurance scheme distinguishes the subsidized regime, financed by the state for low-income households, from the contributory regime, financed by payroll contributions.

Comorbidity burden was summarized by the Charlson index total rather than by individual conditions. This was a deliberate consequence of the parameter budget: entering each comorbid condition separately would have consumed several parameters for a construct that the index already summarizes, at a sample size that does not support the expenditure. The trade-off is that the specific nature of comorbidity is not modeled, a limitation we address explicitly in Section 4.6.

Handgrip strength was measured with a hydraulic hand dynamometer following a standardized protocol of three maximal contractions; the highest value was used. Sex was coded as a binary indicator, and hospitalization during the acute episode served as a proxy for acute-phase severity. Admission to an intensive care unit (ICU) during the index infection was recorded as an additional marker of acute illness severity. This variable was evaluated exclusively within secondary sensitivity analyses (Section 2.7 and Section 3.9).

### 2.4. Sample Size Assessment

We computed the minimum sample size required to develop a prediction model with a binary outcome using the three criteria of Riley et al.: a global shrinkage factor of at least 0.9, an absolute difference no greater than 0.05 between apparent and adjusted Nagelkerke R^2^, and estimation of the overall outcome risk with a margin of error of 0.05 [22,23]. The anticipated Cox–Snell R^2^ was derived from the anticipated C statistic and outcome prevalence [27]. Calculations were implemented in Python 3.13 and are equivalent to the pmsampsize package. The assessment was performed for the 97 candidate parameters originally considered in an earlier version of this work and for intermediate counts, so that the consequence of dimensionality is explicit.

### 2.5. Model Development

Ridge-penalized logistic regression was pre-specified as the primary model on a priori grounds: parsimony, calibration behavior and interpretability at small sample size, together with evidence that ML algorithms do not systematically outperform logistic regression for tabular clinical prediction [28]. It was not selected by comparing cross-validated performance across candidate algorithms because, with approximately 130 observations, such differences fall within sampling noise, and selecting the maximum introduces winner’s-curse bias [29]. Lasso-penalized logistic regression, random forest and gradient boosting were evaluated as pre-specified sensitivity analyses only. Comparing penalized regression against ensemble algorithms under a common interpretability framework follows an approach previously validated by our group for cohort-level prediction from aggregate clinical data [30].

The penalty parameter was tuned by ten-fold internal cross-validation using negative log loss rather than AUC. This choice is deliberate: log loss is a proper scoring rule and therefore rewards discrimination and calibration jointly, whereas tuning on AUC optimizes ranking alone and can select a penalty yielding poorly calibrated probabilities, which would invalidate the decision curve analysis.

Missing predictor values were handled by multivariate imputation by chained equations, fitted inside the modeling pipeline. Continuous predictors were standardized. Every preprocessing step was contained within the pipeline so that imputation, scaling and penalty selection were re-executed within each resampling replicate; fitting any of these outside the resampling loop is the most common route by which an ostensibly optimism-corrected estimate remains optimistic.

No class-imbalance correction was applied. The outcome proportion of 0.70 does not constitute an imbalance problem, and oversampling methods degrade calibration without improving discrimination, with harm increasing at small sample sizes and high dimensionality [25,26]. Where greater sensitivity was required, the decision threshold was varied explicitly instead.

### 2.6. Internal Validation

We did not reserve a hold-out test set. At this sample size, a split design fails twice: it trains the model on fewer observations and estimates performance in a subset so small that the confidence interval is uninformative, since for 26 participants the 95% interval for the AUC spans approximately ±0.20. Instead, the entire cohort was used for development, and optimism was estimated by bootstrap following the Harrell–Efron procedure with 1000 replicates [31,32]. Within each replicate, the complete modeling procedure was re-executed, the resulting model was evaluated on the bootstrap sample and on the original cohort, and optimism was taken as the difference. Corrected estimates are the apparent performance of the final model minus mean optimism; 95% confidence intervals are percentiles of the corrected distribution.

Discrimination was summarized by the AUC, calibration was summarized by the calibration slope and calibration-in-the-large intercept, and overall accuracy was summarized by the Brier score. Calibration was displayed as a locally weighted scatterplot smoothing curve on out-of-fold predictions rather than as a five-bin plot, which, at n = 130, would place approximately 26 observations per bin.

### 2.7. Clinical Utility, Stability, Interpretability and Fairness

Clinical utility was assessed by decision curve analysis over a threshold range of 0.30 to 0.90, justified a priori: below 0.30, nearly every patient would be referred given the observed prevalence, and above 0.90, the model would need near-certainty to withhold referral [31,32,33,34].

While the evaluation range was defined *a priori*, the operational decision threshold was identified empirically as the point of maximum incremental net benefit within out-of-fold predictions. To account for potential selection optimism arising from data-driven threshold identification, we performed a nested bootstrap procedure (1000 replicates). Within each resample, the optimal threshold was re-identified, and net benefit was evaluated on both the resampled (apparent) and original cohorts to estimate optimism (mean difference) and quantify threshold selection uncertainty across replicates (Section 3.4).

Prediction stability was quantified by refitting the model on 500 bootstrap resamples and predicting the original cohort each time, following Riley and Collins [35]. We report the mean absolute prediction error per participant and the proportion of resamples in which a participant’s classification changed at the selected threshold.

Model explanation used SHapley Additive exPlanations (SHAP) computed on out-of-fold predictions, so that each explanation derives from a model that had not seen that participant. Because importance rankings are themselves unstable at small sample size, we report the bootstrap distribution of each predictor’s rank across 200 resamples rather than a single ordered list.

Feature contributions were further evaluated through iterative model ablation across all eight predictors using ten-fold cross-validation of the full pipeline. Changes in out-of-fold AUC and Brier scores were quantified against the complete specification, deriving 95% confidence intervals via paired bootstrap resampling with 2000 replicates (Section 3.5, Supplementary Table S9).

Fairness was assessed on out-of-fold predictions across the full cohort, not a hold-out subset, for sex, socioeconomic stratum, insurance type and educational level. We report subgroup AUC with bootstrap confidence intervals, demographic parity difference (DPD) and equalized odds difference (EOD), with a pre-specified tolerance of 0.10 and a minimum subgroup size of 20.

Estimation uncertainty for pairwise DPD and EOD was quantified via 1000 stratified bootstrap resamples across demographic subsets (Section 3.7, Supplementary Table S1). Furthermore, a dedicated sensitivity model assessed hospitalization effect stability by adjusting for acute ICU admission as an exploratory marker of severe acute illness (Section 3.9, Supplementary Table S11), noting that elapsed time since infection remained unrecorded (Section 2.1 and Section 4.6).

### 2.8. Secondary Ordinal Analysis

Because dichotomizing a five-level scale discards information the cohort can ill afford to lose, we fitted a proportional-odds cumulative logit model on the full PCFS grade using the same eight predictors [36]. Discrimination was summarized by a generalized ordinal concordance index, which reduces exactly to the AUC for a two-level outcome, with the same bootstrap optimism correction. The proportional-odds assumption was examined descriptively by comparing predictor coefficients across cumulative cut-points [37].

### 2.9. Reporting, Risk of Bias and Reproducibility

Reporting followed TRIPOD + AI [19], and risk of bias was self-assessed using PROBAST + AI [21] (See Tables S6 and S7); both completed instruments are provided as Supplementary Materials. Analyses used Python 3.12 with scikit-learn, statsmodels and SHAP. The complete analysis code, including a synthetic cohort generator that allows the entire pipeline to be executed without participant data, is publicly available (Section Data Availability). All reported values are generated programmatically into a machine-readable results file; no figure in this manuscript was transcribed by hand.

Supplementary and sensitivity analyses were executed using identical random seeds within the original version-controlled pipeline to guarantee consistency across out-of-fold predictions. Methodologically, the eight primary predictors, the ridge-penalized model, the [0.30, 0.90] threshold range, the bootstrap optimism correction, the SHAP analysis, the ordinal model, and the fairness parameters were strictly pre-specified before modeling. In contrast, the operating threshold selection, model-ablation analysis, fairness-parity confidence intervals, and ICU-adjusted hospitalization model (Nested-Bootstrap Uncertainty Around the Operating Threshold Section and Model-Ablation Analysis Section and Section 3.7 and Section 3.9) were conducted as post hoc exploratory assessments to quantify parameter uncertainty and robustness.

## 3. Results

### 3.1. Cohort Characteristics

Of 130 participants with complete outcome ascertainment, 91 (70.0%) reported some degree of functional limitation (PCFS grades 1–4), and 39 (30.0%) reported none. Missing data among the eight pre-specified predictors were uniform at 6.5% and attributable entirely to the nine records excluded for absent outcome, leaving no missingness within the analytic sample (Supplementary Table S5). Table 1 presents the cohort stratified by functional status. Disaggregating the binary outcome revealed 39 participants at grade 0, 33 at grade 1, 45 at grade 2, 12 at grade 3, and one at grade 4 (Supplementary Table S8). Most participants with functional impairment clustered in grades 1 and 2 (78/91, 85.7%), with only one individual in grade 4. Results from the secondary proportional-odds model (Section 3.8, Supplementary Table S3) demonstrated consistent predictor ordering, confirming that the primary dichotomization retained key multivariable patterns without loss of predictive hierarchy.

Three variables differed materially between groups. MFIS total score was almost three times higher in participants with limitation (median 26.0, interquartile range 16.0–40.5) than in those without (9.0, 6.0–25.0; *p* < 0.001), the largest separation observed for any variable. PSQI global score was also higher in the limitation group (7.0, 4.0–12.0 versus 4.0, 2.0–7.5; *p* < 0.001). Hospitalization during the acute episode showed the opposite direction to that anticipated: 53.8% of participants without limitation had been hospitalized compared with 26.4% of those with limitation (*p* = 0.005).

Maximum grip strength was lower in participants with limitation (median 23.8 kg, 17.8–30.2) than in those without (26.0 kg, 21.3–35.4), but the difference did not reach conventional significance (*p* = 0.056). Age, MoCA total, Charlson index, sex, socioeconomic stratum, insurance type, educational level and ethnicity did not differ appreciably. The comorbidity profile of the cohort was light: the median Charlson index was 0 overall and 0 with an interquartile range of 0–0 in participants without limitation, indicating that at least three quarters of that group carried no scored comorbidity, with an upper quartile of 3 in the limitation group. This distribution constrains the discriminatory capacity of comorbidity in this sample and should be borne in mind when interpreting its modest contribution to the model. These univariable comparisons are descriptive; predictor selection was pre-specified and did not depend on them, and no variable was included or excluded on the basis of a *p* value.

**Figure 1:**
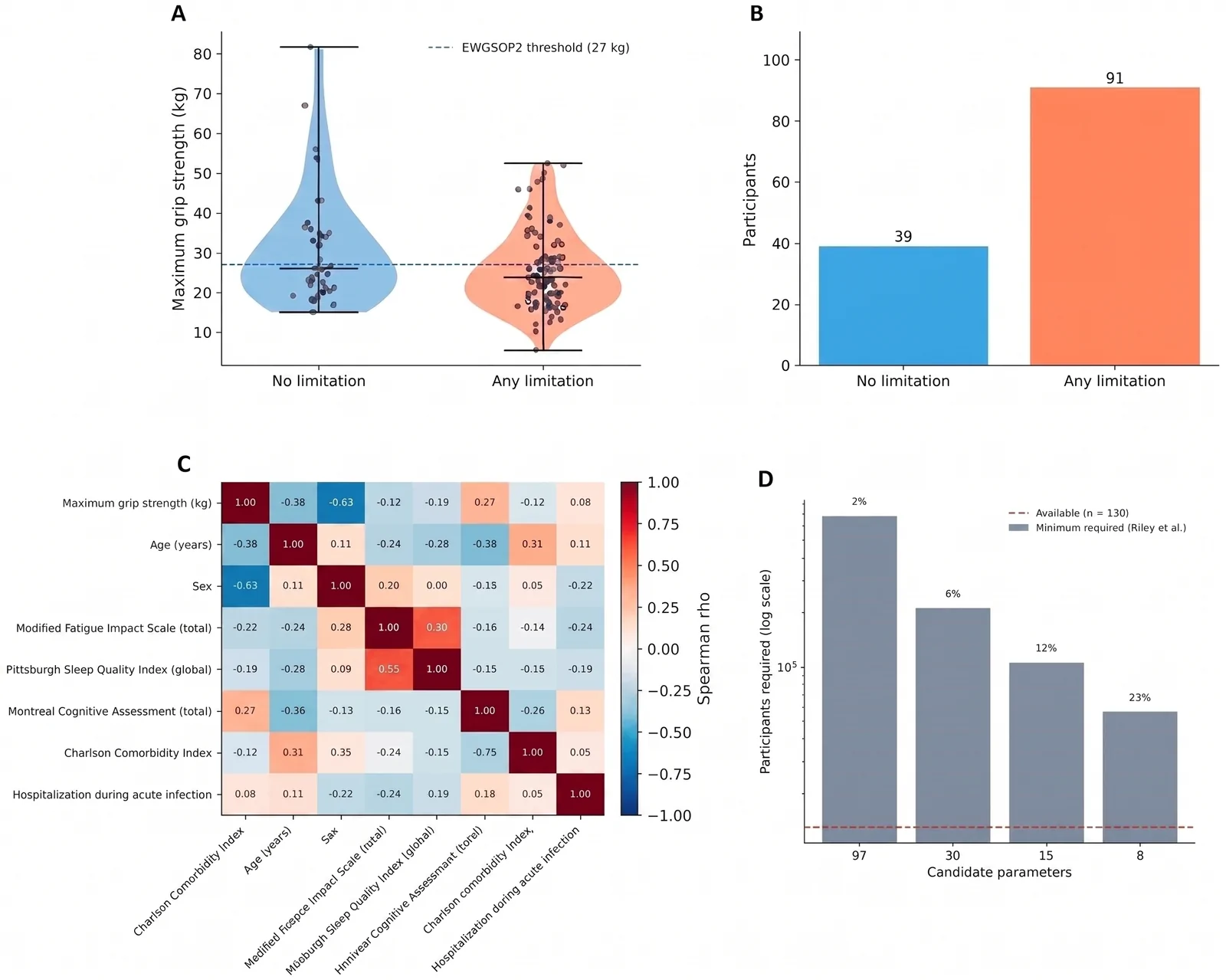
Exploratory characterization of the study population (n = 130). (**A**) Maximum handgrip strength by functional status, with individual observations overlaid and the EWGSOP2 reference threshold (27 kg) indicated; (**B**) distribution of the dichotomized outcome; (**C**) Spearman correlation matrix restricted to the eight pre-specified predictors; (**D**) minimum sample size required for model development according to the criteria of Riley et al. across candidate-parameter counts (logarithmic scale), with the available sample size indicated and the percentage of the requirement met annotated above each bar. Abbreviations: EWGSOP2, European Working Group on Sarcopenia in Older People 2. Source: own elaboration.

Figure 1 characterizes the cohort visually. Panel A shows substantially overlapping grip strength distributions, with the median of both groups near or below the European Working Group on Sarcopenia in Older People (EWGSOP2) reference threshold of 27 kg, indicating that low grip strength was common irrespective of functional status. Panel B displays the outcome distribution. Panel C shows that the strongest correlation among pre-specified predictors was between MFIS and PSQI (Spearman rho = 0.58), consistent with fatigue and sleep disturbance forming a partially shared construct; grip strength correlated moderately with sex (rho = −0.63) and inversely with age (rho = −0.38), as expected physiologically, but only weakly with MFIS (rho = −0.22). Panel D presents the sample size assessment described next.

### 3.2. Sample Size Assessment Determines the Framing of the Analysis

Table 2 reports the minimum sample size required under the criteria of Riley et al. Assuming the observed outcome proportion of 0.70 and an anticipated C statistic of 0.715, the shrinkage criterion dominated in every scenario. Developing a model with the 97 candidate parameters considered in an earlier version of this work would require 6855 participants and 2057 events; restricting to 15 parameters would require 1060, and restricting to the eight parameters used here would require 566. With 130 participants available, the cohort satisfies 23.0% of the requirement for the final model and 1.9% of the requirement for the original candidate set.

This result determined the framing of the entire analysis. The available sample falls below the minimum required for confirmatory model development under all three criteria (Table 2). We therefore report all subsequent findings as exploratory and hypothesis-generating, avoid any language of validation or deployment, and present the sample size table as a primary result rather than a methodological footnote. Each subsequent analysis is accordingly reported together with an explicit measure of its own uncertainty rather than as a point estimate: bootstrap rank intervals for the SHAP ordering, confidence intervals for the ablation differences, bootstrap confidence intervals for the parity metrics, and nested-bootstrap uncertainty for the operating threshold. These are presented so that the precision supported by this sample can be assessed directly, not as evidence that the sample is adequate.

### 3.3. Model Performance and Calibration

Table 3 compares the four candidate algorithms on identical out-of-fold predictions, together with a no-skill reference. The pre-specified ridge model achieved a cross-validated AUC of 0.728 (95% CI 0.625–0.823). Lasso, random forest and gradient boosting achieved 0.723, 0.718 and 0.719 respectively, all with widely overlapping intervals. Paired bootstrap comparisons of the AUC difference against the ridge model included zero in every case (lasso +0.004, 95% CI −0.013 to +0.023; random forest +0.010, −0.033 to +0.053; gradient boosting +0.008, −0.068 to +0.077). We therefore make no claim that any algorithm outperformed another, and the pre-specification of ridge regression rests on parsimony and calibration rather than on measured superiority.

Calibration discriminated between the algorithms more clearly than did the AUC. The ridge model achieved a calibration slope of 0.907 and intercept of 0.002, and random forest 0.954 and −0.001, both close to ideal. Gradient boosting, by contrast, produced a slope of 0.486 with intercept −0.103, indicating markedly overconfident predictions. This dissociation illustrates why discrimination alone is an insufficient basis for model choice when predicted probabilities are intended to inform a threshold-based decision.

The no-skill reference row merits explicit attention. A model that assigns every participant the cohort prevalence attains an F1 score of 0.824 and positive predictive value of 0.700, both higher than any real model in the table, purely because the outcome occurs in 70% of participants. Reporting F1 or positive predictive value without this comparator would misrepresent model performance in either direction, and we recommend that studies at similar prevalence include it routinely.

**Figure 2:**
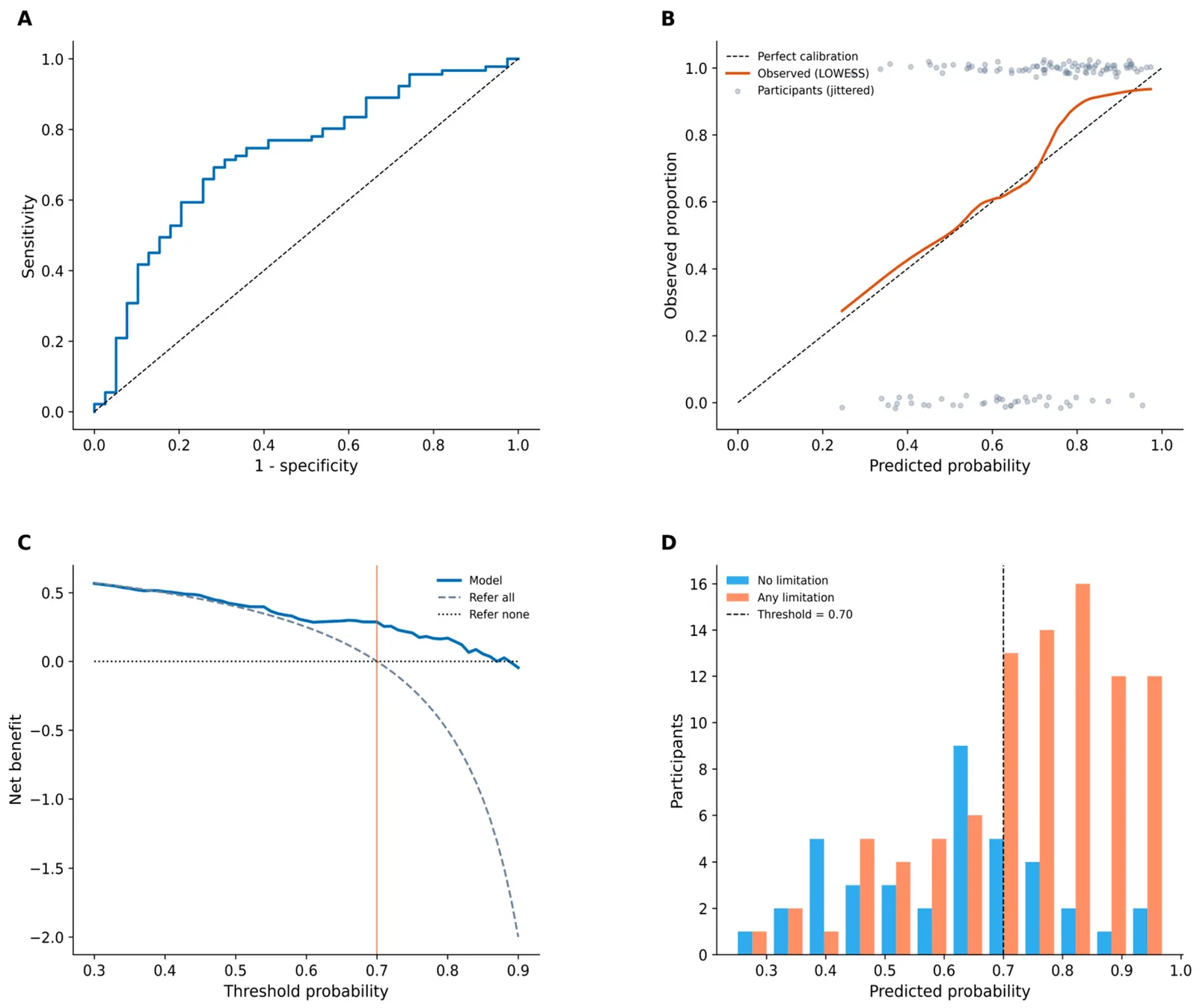
Discrimination, calibration and clinical utility of the primary model. (**A**) Receiver operating characteristic curve on out-of-fold predictions; (**B**) flexible calibration curve estimated by locally weighted scatterplot smoothing, with individual participants shown as jittered points; (**C**) decision curve analysis comparing the model against the strategies of referring all and referring no patients, with the selected operating threshold indicated; (**D**) distribution of predicted probabilities stratified by observed outcome, with the selected threshold indicated. Source: own elaboration.

Bootstrap internal validation of the primary model, applying the full pipeline within each of 1000 replicates, is summarized in Table 3 and Figure 2. The apparent AUC of 0.794 fell to an optimism-corrected 0.748 (95% CI 0.669–0.830), an optimism of 0.046. This value is reported descriptively and should not be read as evidence against overfitting: low measured optimism is consistent with a penalized model of limited effective complexity, but at this sample size, the bootstrap has limited capacity to detect overfitting, and the confidence intervals around the corrected estimates remain wide. The prediction-stability analysis in Section 3.6 is the more informative assessment of this question and points in a less favorable direction. The optimism-corrected Brier score was 0.185 (0.149–0.230), against 0.210 for the no-skill reference.

Calibration after optimism correction was acceptable. The calibration slope was 1.15 (95% CI 0.71–1.69), and the calibration-in-the-large intercept was −0.034 (−0.498 to 0.399); both intervals include their ideal values of 1 and 0 respectively. Figure 2B shows the flexible calibration curve tracking the identity line closely between predicted probabilities of approximately 0.45 and 0.70, with modest overestimation of risk at the lower extreme, where few participants were observed. That calibration is demonstrable at all is a direct consequence of omitting oversampling; the wide confidence interval around the slope, however, reflects the sample size and should temper any interpretation of the point estimate.

Figure 2A presents the receiver operating characteristic curve, and Figure 2D presents the distribution of predicted probabilities by observed outcome, which shows separation of the two distributions with substantial overlap between predicted probabilities of 0.55 and 0.75, the region where most misclassification occurred.

### 3.4. Clinical Utility

Decision curve analysis (Figure 2C) demonstrated a net benefit exceeding both default strategies, referring to all patients and referring none, across threshold probabilities from 0.38 to 0.88. The maximum incremental net benefit was 0.287 at a threshold of 0.70, which was therefore adopted as the operating point for all threshold-dependent metrics. At this threshold, the model would refer 74 of 130 participants and, relative to referring everyone, avoids approximately 12.3 unnecessary referrals per 100 patients assessed without missing additional cases.

**Figure 3:**
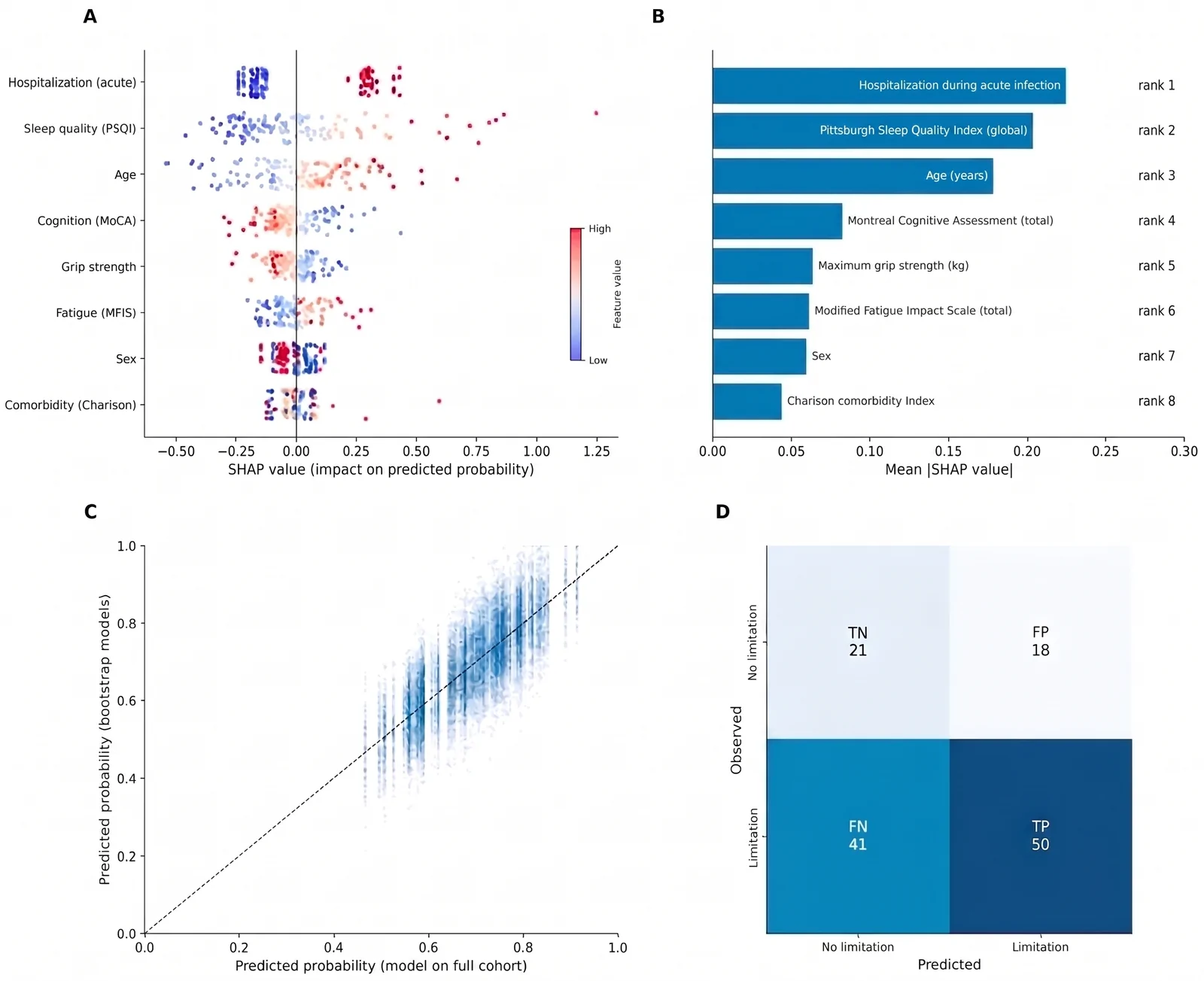
Model interpretability and stability. (**A**) SHAP summary (beeswarm) of out-of-fold predictions, in which each point represents one participant and color encodes that participant’s value of the predictor; (**B**) mean absolute SHAP value per predictor, annotated with the 95% bootstrap interval of each predictor’s importance rank across 200 resamples; (**C**) individual-level prediction instability, plotting the prediction of the model developed on the full cohort against predictions from models refitted on 500 bootstrap resamples; (**D**) confusion matrix at the pre-specified threshold of 0.70. Abbreviations: FN, false negative; FP, false positive; SHAP, SHapley Additive exPlanations; TN, true negative; TP, true positive. Source: own elaboration.

Classification performance at this threshold was as follows: sensitivity 0.692 (95% CI 0.591–0.778), specificity 0.718 (0.562–0.835), positive predictive value 0.851 (0.753–0.915) and negative predictive value 0.500 (0.373–0.627). The confusion matrix (Figure 3D) comprised 63 true positives, 28 false negatives, 28 true negatives and 11 false positives. The negative predictive value of 0.500 is the operationally limiting figure: among participants the model classified as unlikely to have functional limitation, half in fact had it. Any prospective use would therefore need to be as a triage aid that supplements rather than replaces clinical assessment, and this constraint is more informative about clinical readiness than the AUC.

That net benefit is positive across a wide and clinically plausible threshold range is the substantive answer to whether discrimination of this magnitude is meaningful. It is nonetheless a within-sample result and does not establish that acting on the model would improve outcomes.

#### Nested-Bootstrap Uncertainty Around the Operating Threshold

Quantification of selection optimism via nested bootstrap resampling (1000 replicates) demonstrated stable operating threshold identification, with a resampled median threshold of 0.71 (95% CI 0.66–0.80; range 0.54–0.84). Mean optimism in net benefit attributable to threshold derivation was 0.036. Consequently, the selection-corrected net benefit at $p_{t}=0.70$ was estimated at 0.254 (95% CI 0.152–0.287), contextualizing the apparent value of 0.287 (Supplementary Table S10).

### 3.5. Relative Influence of Predictors

Table 4 reports odds ratios per standard deviation from the final ridge model. MFIS had the largest coefficient (odds ratio 1.62 per standard deviation), followed by PSQI (1.40) and the Charlson index (1.28). Grip strength was associated with lower odds of limitation (0.86), in the anticipated protective direction, but ranked fifth in absolute magnitude. Hospitalization during the acute episode showed the strongest inverse association (0.72). Coefficients are on the standardized scale and therefore express change per standard deviation, not per raw unit; because the model is penalized, they are shrunk toward zero and should not be interpreted as unbiased causal effect estimates.

SHAP analysis on out-of-fold predictions (Figure 3A,B; Supplementary Table S2) reproduced this ordering. MFIS had the highest mean absolute SHAP value (0.402) and ranked first in 54.0% of 200 bootstrap resamples, with a rank interval of 1 to 5. Hospitalization ranked second (0.320; first in 26.0% of resamples), and PSQI ranked third (0.291; 16.0%). Grip strength ranked fifth by point estimate (0.121), with a median bootstrap rank of 6, a rank interval spanning 2 to 8, and first place in only 0.5% of resamples. MoCA and sex contributed least.

Two features of this result require emphasis. First, the ordering of the leading three predictors is comparatively robust, whereas the ordering within the lower half of the ranking is largely indistinguishable from noise, since grip strength, age, MoCA and sex all have rank intervals extending to position 8. Reporting a single ordered importance list without these intervals would imply a precision the data do not support. Second, SHAP quantifies each variable’s contribution within the fitted model; it is not evidence of biological or causal relevance, and the influence of a variable may reflect its correlation with unmeasured factors.

The beeswarm plot (Figure 3A) adds directional information. Higher MFIS and PSQI values shifted predictions toward limitation, as expected. For hospitalization, the pattern was clearly bimodal and inverse: hospitalized participants received predictions shifted away from limitation. For grip strength, higher values shifted predictions away from limitation, consistent with the univariable direction, but with contributions concentrated near zero.

#### Model-Ablation Analysis

Predictor contribution robustness was evaluated by iteratively refitting the full cross-validation pipeline across single-variable omissions (Section 2.7). No predictor’s removal produced a statistically significant change in discrimination: the 95% confidence interval for the difference in out-of-fold AUC included zero for all eight predictors (Supplementary Table S9). The analysis therefore functions as a consistency check on the direction and relative magnitude of the SHAP ordering, not as a significance test of predictor importance. In agreement with SHAP rankings, excluding MFIS yielded the largest reduction in out-of-fold discrimination (AUC 0.6816 versus 0.7275; ΔAUC = 0.046, 95% CI −0.001 to 0.094), followed by hospitalization (AUC 0.6940; ΔAUC = 0.035, 95% CI −0.013 to 0.082) and PSQI (AUC 0.7078; ΔAUC = 0.020, 95% CI −0.011 to 0.053). In contrast, omitting grip strength yielded no performance penalty (AUC 0.7374; ΔAUC = −0.010, 95% CI −0.028 to 0.007), with minor shifts observed for remaining covariates. Read with the caveat above, the pattern of point estimates is consistent with self-reported fatigue accounting for the largest share of predictive capability in this cohort and with grip strength contributing none that the remaining predictors do not already supply (Supplementary Table S9).

ΔAUC=0.046−0.0010.094ΔAUC=0.035ΔAUC=0.020ΔAUC=−0.010−0.0280.007

### 3.6. Prediction Stability

Refitting the model on 500 bootstrap resamples (Figure 3C) showed a mean absolute prediction error of 0.071 per participant and a median of 0.069, with a maximum of 0.166. Mean classification instability at the 0.70 threshold was 0.131, and 39 of 130 participants (30.0%) changed classification in more than 20% of resamples.

The model is reasonably stable in aggregate, as the bootstrap cloud in Figure 3C is centered on the identity line, but individual-level predictions are not yet dependable: for almost one-third of participants, whether the model recommends referral depends materially on which resample of the same 130 individuals was used for fitting. This finding is consistent with, and quantifies, the sample size shortfall identified in Section 3.2 and constitutes the principal reason why the model should not be used for individual decisions.

### 3.7. Exploratory Fairness Assessment

**Figure 4:**
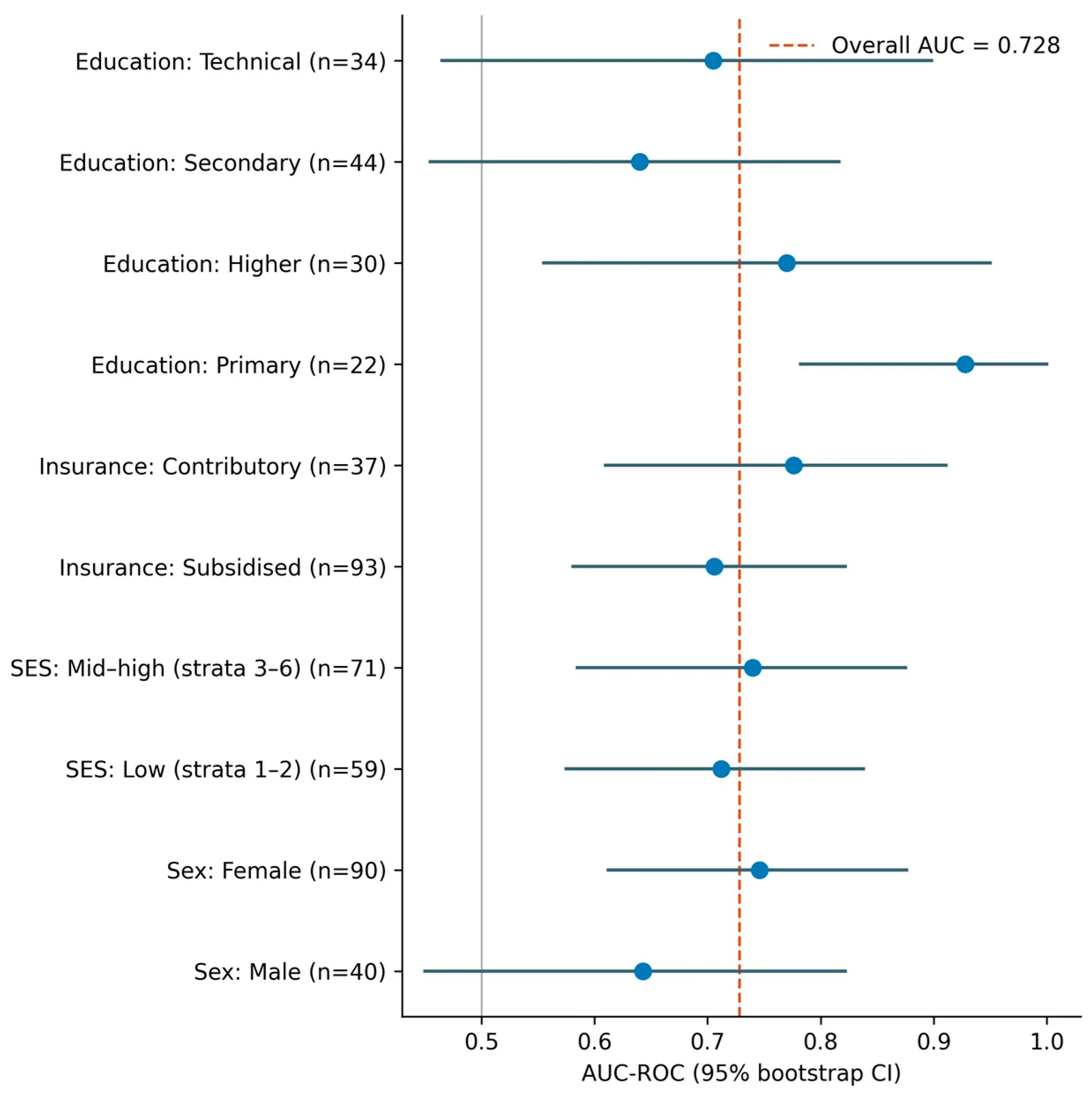
Exploratory subgroup discrimination across sociodemographic strata. Point estimates of the area under the receiver operating characteristic curve with 95% bootstrap confidence intervals, computed on out-of-fold predictions across the full cohort. The dashed vertical line indicates overall discrimination, and the solid vertical line indicates chance-level performance. Subgroup sizes are shown in parentheses; subgroups with fewer than 20 participants were excluded. Wide and overlapping intervals preclude inference of differential performance. Source: own elaboration.

Subgroup performance across the full cohort is shown in Figure 4, and parity metrics are shown in Supplementary Table S1. Overall out-of-fold AUC was 0.728. Discrimination varied across subgroups, but confidence intervals were wide and overlapped substantially in every comparison: sex 0 achieved 0.643 (95% CI 0.451–0.821) against 0.746 (0.612–0.875) for sex 1; low socioeconomic stratum 0.712 (0.574–0.837) against 0.739 (0.584–0.874) for mid-to-high stratum. The apparently high AUC of 0.927 for participants with primary education rests on 22 individuals and has a lower confidence bound of 0.781; it should not be interpreted as superior performance in that group.

Parity metrics exceeded the pre-specified tolerance of 0.10 in several comparisons. For sex, DPD was 0.281, and EOD was 0.318, both above tolerance. Insurance type also exceeded both thresholds (DPD 0.116; EOD 0.266). Among educational strata, EOD exceeded tolerance in six of nine pairwise comparisons, reaching 0.462 between primary and secondary education. Socioeconomic stratum was the only attribute for which neither metric exceeded tolerance (DPD 0.080; EOD 0.066).

Bootstrap 95% confidence intervals highlighted substantial variation in estimation precision across sociodemographic metrics (Supplementary Table S1). Parity metrics across sexes showed robust differences, with DPD (95% CI 0.11–0.45) and EOD (95% CI 0.13–0.53) intervals clearly excluding zero. Conversely, comparisons across socioeconomic strata (DPD 0.080; 95% CI 0.004–0.26) and educational levels displayed wide intervals extending well above the 0.10 tolerance threshold, reflecting the small size of individual subgroups. These findings indicate that sex disparity was the only consistently supported divergence, whereas other subgroup metrics reflect exploratory uncertainty.

These disparities are reported as exploratory findings requiring confirmation, not as established algorithmic bias. Several considerations constrain their interpretation: subgroups contain between 22 and 93 participants; DPD ignores the outcome and may reflect genuine differences in underlying risk rather than unfair treatment; and the number of pairwise comparisons performed without multiplicity adjustment makes some exceedances expected by chance. The direction of the sex disparity nonetheless aligns with the differing outcome prevalence between sex strata (0.600 versus 0.744) and warrants examination in larger samples.

### 3.8. Secondary Ordinal Analysis

The proportional-odds model using all five PCFS grades achieved an optimism-corrected ordinal concordance index of 0.734 (95% CI 0.679–0.784), closely comparable to the binary AUC of 0.748, with a McFadden pseudo-R^2^ of 0.155 (Supplementary Table S3). The ordering of predictors was preserved: MFIS had the largest odds ratio per standard deviation (2.87), followed by age (1.65) and the Charlson index (1.33), with grip strength (0.79) and hospitalization (0.63) inversely associated. Seven of eight predictors agreed in direction with the binary model; sex and MoCA, the two smallest contributors, were the exceptions.

Using the full ordinal scale therefore did not improve discrimination, but it did not degrade it either, and it corroborates the primary finding that fatigue rather than grip strength dominates. The proportional-odds assumption was examined across cumulative cut-points (Supplementary Table S4). The cut-point separating grade 4 from lower grades is not interpretable because only one participant had grade 4, and its coefficients were correspondingly unstable. Restricted to the three estimable cut-points, coefficient variation was modest for most predictors, namely 0.10 for hospitalization, 0.15 for the Charlson index, 0.32 for grip strength and 0.61 for MFIS, supporting the common-slope assumption for those variables, with greater variation for sex (1.12) and PSQI (0.94). We report this assessment as descriptive rather than as a formal test.

### 3.9. Sensitivity Analysis: Hospitalization Adjusted for ICU Admission

A multivariable sensitivity analysis incorporating acute ICU admission alongside the primary predictors assessed the robustness of the inverse hospitalization association. Adjustment for ICU status yielded an essentially identical hospitalization coefficient (odds ratio 0.71 vs. 0.72; Table 5), with ICU admission demonstrating a negligible effect (odds ratio 1.07) and low overlap (five of 45 hospitalized participants admitted to ICU). These results indicate that acute critical illness does not explain the observed association, consistent with potential ascertainment or referral patterns (Section 4.3), although unrecorded time since infection precludes complete temporal evaluation (Section 2.1 and Section 4.6; Supplementary Table S11).

## 4. Discussion

### 4.1. Principal Findings

To our knowledge, this is among the first analyses to test handgrip strength formally against fatigue, sleep quality, cognition and acute-phase severity within a single pre-specified model in a Latin American post-COVID cohort and among very few post-COVID prediction studies to report discrimination, calibration, clinical utility and prediction stability jointly rather than discrimination alone. Three findings stand out. Fatigue, measured by the MFIS, was consistently the most influential correlate of functional limitation, ranking first in more than half of bootstrap resamples. Handgrip strength, the variable that motivated the original study hypothesis, ranked fifth of eight, with a rank interval spanning positions 2 to 8, and did not reach conventional significance in univariable comparison. Hospitalization during the acute episode was inversely associated with functional limitation, a pattern with recent precedent in the literature (Section 4.3). Discrimination after optimism correction was moderate (AUC 0.748), calibration was acceptable, and net benefit was positive across a wide threshold range; the principal constraint, addressed directly rather than left implicit, is that individual-level predictions were unstable for approximately 30% of participants (Section 3.6). Sensitivity analyses evaluating predictor ablation, decision threshold optimism, fairness parity confidence intervals, and hospitalization adjustment for ICU admission (Nested-Bootstrap Uncertainty Around the Operating Threshold Section and Model-Ablation Analysis Section and Section 3.7 and Section 3.9) reinforced the main conclusions. While quantifying selection optimism and parameter uncertainty across subgroups, these additional evaluations did not alter the relative importance of the identified correlates.

### 4.2. Fatigue Rather than Muscle Strength

Our finding that fatigue dominates is concordant with, and extends, the broader post-COVID literature. Fatigue is the most prevalent persistent symptom across cohorts, reported by 57% of participants in a large Mexican cross-sectional study and 70% in a German prospective cohort [4,16]. In the largest long-term follow-up of post-COVID recovery to date, a four-year prospective cohort of 3590 individuals, fatigue severity was the strongest predictor of failure to recover, with a recovery hazard ratio of 0.37 for high fatigue, larger in magnitude than myalgia or dysautonomic symptoms; the authors explicitly recommend that research and clinical services prioritise fatigue severity as the strongest predictor of non-recovery [38]. Hierarchical modeling in an earlier cohort attributed 51.3% of the variance in functional limitation to fatigue, dizziness and memory loss combined, with fatigue carrying the largest individual coefficient [14]. Our result, obtained through an independent modeling approach in an independent population, converges on the same ordering.

The relative demotion of grip strength deserves careful interpretation because it does not contradict prior work so much as reframe it. Studies reporting associations between low handgrip strength and worse post-COVID functional outcomes have generally examined strength in isolation or adjusted for demographic covariates only [11]. Our analysis indicates that much of that association is shared with fatigue and sleep disturbance once all three are modeled jointly: grip strength retained a coefficient in the expected protective direction (odds ratio 0.86 per standard deviation) but contributed less than fatigue. This interpretation is supported by the model-ablation analysis (Model-Ablation Analysis Section), in which omitting grip strength did not compromise out-of-fold discrimination, whereas omitting the MFIS produced the only performance reduction approaching statistical significance. This is consistent with mechanistic evidence that reduced strength in post-COVID syndrome is mediated substantially by limb muscle mass [12] and with the finding that a single maximal contraction is a limited summary of the underlying pathology: multidimensional grip protocols capturing fatigability and recovery capacity discriminate patients from recovered controls considerably better than peak force alone [8,17]. Appelman and colleagues demonstrated that muscle abnormalities worsen specifically after post-exertional malaise [39], and a 2025 one-year follow-up study likewise found that perceived fatigue, rather than baseline strength, tracked the slower recovery trajectory in more severely affected patients [40]. Together, this evidence points to dynamic intolerance to exertion, rather than static weakness, as the clinically relevant muscular phenomenon, precisely the construct that the MFIS captures and that peak grip strength does not.

A distributional feature of our cohort reinforces this reading. Median grip strength was at or below the EWGSOP2 reference threshold of 27 kg in both outcome groups (Figure 1A), so low grip strength was near-ubiquitous in this sample and had correspondingly limited capacity to discriminate between groups. In cohorts with greater variance in strength or enriched for older or previously hospitalized patients, grip strength may well perform differently, and this is a boundary condition of our finding rather than a rebuttal of the physiological literature.

Sleep quality emerged as the second most influential self-reported measure, with a PSQI odds ratio of 1.40 per standard deviation. The moderate correlation between MFIS and PSQI in our data (rho = 0.58) indicates that fatigue and sleep disturbance are partially overlapping but not redundant constructs, and both retained independent contributions, consistent with cohort evidence linking sleep disturbance to persistent post-COVID symptom burden [16]. This joint contribution suggests that interventions targeting sleep merit evaluation alongside graded activity approaches in rehabilitation programs [15,16].

### 4.3. The Inverse Association with Hospitalization

Hospitalized participants had lower odds of functional limitation in our data, a direction that runs counter to the general expectation that greater acute severity predicts worse long-term outcomes. This pattern, while counterintuitive, is not isolated. A 2025 multicenter prospective cohort of 208 patients found that participants with severe acute COVID-19 reported significantly lower long-term fatigue and higher health-related quality of life than those with mild or moderate acute disease, describing the result as challenging the assumption that greater illness severity leads to more long-term impairment [41]. Our finding sits within this emerging pattern rather than standing apart from it.

A multivariable sensitivity analysis incorporating ICU admission as an independent marker of acute-phase severity (Section 3.9) yielded an essentially unchanged hospitalization odds ratio (0.71 vs. 0.72) and a near-null association for ICU status (odds ratio 1.07), with minimal overlap between both indicators (five of 45 hospitalized participants admitted to the ICU). These findings indicate that the inverse hospitalization association is not driven by undifferentiated acute illness severity within the inpatient subgroup. However, potential confounding attributable to elapsed time between acute infection and assessment could not be evaluated due to unrecorded assessment dates (Section 4.6), representing an intrinsic limitation of the available cohort data.

Three mechanisms could plausibly generate this direction, and they are not mutually exclusive. Hospitalized patients in this health system may have received early mobilization and structured rehabilitation referral that non-hospitalized patients did not; work in Colombian intensive care settings found that mortality was associated with clinical and ventilatory management rather than with socioeconomic position or insurance scheme, indicating that what happens during admission carries measurable weight in this system [7]. A survivorship effect is also plausible: participants hospitalized with the most severe disease and the greatest functional damage may have been less likely to attend outpatient follow-up. Most plausibly in our view, a referral and ascertainment pathway effect may operate: non-hospitalized individuals who nonetheless sought post-COVID care are, by construction, those whose persistent symptoms were troublesome enough to prompt consultation, whereas hospitalized individuals may have entered follow-up automatically regardless of symptom burden, so that hospitalization status acts partly as a marker of route into the cohort rather than of biological severity.

These mechanisms cannot be distinguished with cross-sectional data from a single care pathway, and we report the association, together with its recent precedent, rather than interpreting it causally. Resolving it requires cohorts that enroll at the point of acute infection rather than at the point of post-COVID consultation.

### 4.4. Performance in Context

An optimism-corrected AUC of 0.748 compares favorably with the published post-COVID prediction literature. Models predicting long COVID from acute clinical and immunological data report median AUC values of 0.64 to 0.66 [18], and the Long COVID Computational Challenge, drawing on data from more than 75 institutions, concluded that the binding constraints on clinical adoption are interpretability and workflow integration rather than discrimination [19]. Our estimate is therefore consistent with, rather than an outlier against, an emerging picture in which post-COVID functional trajectories are difficult to predict from routinely available variables, and it indicates that studies in this field reporting substantially higher discrimination from small samples without optimism correction warrant scrutiny for optimism.

The comparison across our own candidate algorithms illustrates why calibration must be reported alongside discrimination, an approach we previously applied when comparing regularized and ensemble models for cohort-level cardiovascular outcomes [30]. Our gradient boosting sensitivity model achieved an AUC statistically indistinguishable from the ridge model while producing a calibration slope of 0.486, predictions too extreme to support a threshold-based referral decision. Selecting on discrimination alone, as remains common practice, would have judged these models equivalent while missing that one of them was unfit for the intended purpose.

### 4.5. Methodological Implications for Small-Sample Prediction Studies

This work illustrates several methodological points of general applicability. The formal sample size assessment showed that the cohort met 23.0% of the requirement for an eight-parameter model and 1.9% for the 97-parameter candidate set originally considered, figures that determined the framing of this study rather than merely qualifying it. Given that 73% of published prediction model studies fall below the minimum requirement and only 8% consider sample size at all [24], reporting this calculation, including when the answer is unfavorable, converts a hidden weakness into an explicit and citable design decision.

The stability analysis quantified overfitting directly rather than discussing it in the abstract. That 30% of participants changed classification across resamples of the same cohort is a more direct and interpretable statement about model reliability than any summary metric, and it is available at no additional data cost [35]; we recommend its routine inclusion in small-sample prediction studies. Reporting subgroup performance on out-of-fold predictions across the full cohort, rather than within an underpowered hold-out, similarly follows recent calls to treat fairness assessment as an integral part of model evaluation rather than an afterthought [42].

Finally, omitting the class-imbalance correction applied in early drafts of this work is worth foregrounding because the practice remains widespread despite the evidence against it. With an outcome proportion of 0.70, there was no imbalance to correct, and simulation evidence shows that oversampling degrades calibration without improving discrimination, with harm increasing as sample size falls [25,^26^]. The calibration we are able to report in Table 4 and Figure 2B is a direct consequence of that decision. A related methodological consideration involves the determination of the operational decision threshold. Because identifying the threshold of maximum net benefit from model predictions introduces selection optimism analogous to hyperparameter tuning, nested bootstrap resampling was implemented to quantify this parameter uncertainty (Nested-Bootstrap Uncertainty Around the Operating Threshold Section). Clinical prediction studies that derive and evaluate decision thresholds on the same sample benefit from reporting this selection uncertainty alongside standard optimism corrections for discrimination and calibration (Section 2.6).

### 4.6. Strengths and Limitations

This analysis combines several methodological features that are individually recommended but rarely reported together in post-COVID prediction studies of comparable size. Predictors were pre-specified in a version-controlled configuration file with a documented clinical rationale for each, so the claim of pre-specification is verifiable rather than asserted. Internal validation re-executed the entire modeling pipeline, imputation, standardization and penalty tuning included, within each of 1000 bootstrap replicates, so the optimism estimate reflects every analytic choice rather than the model fit alone. Discrimination, calibration, clinical utility, prediction stability and subgroup fairness are reported jointly, each with confidence intervals, which allowed us to identify that a competing algorithm with equivalent discrimination had unusable calibration, a distinction that discrimination-only reporting would have missed entirely [24].

Several limitations bound the interpretation of these strengths. The sample size falls well short of the requirement for confirmatory model development, which is why the analysis is presented as hypothesis-generating throughout. The cross-sectional design precludes causal inference. Recruitment from a single municipality in Valle del Cauca, with an ethnic composition in which 66.2% of participants self-identified as being of mixed Indigenous and European ancestry and a socioeconomic distribution concentrated in strata 2 and 3, limits generalizability to settings with different demographic or healthcare characteristics. External validation was not attempted: a suitable independent cohort with concurrent grip dynamometry, MFIS, PSQI, MoCA and PCFS grading in a comparable population is not, to our knowledge, currently available, and validating a model whose individual-level predictions are already unstable would in any case be difficult to interpret; Section 4.7 specifies the sample size such a study would require. Comorbidity was summarized by the Charlson index total rather than by individual conditions, so effects of specific diagnoses relevant to functional recovery cannot be excluded. Fatigue, sleep quality and cognition were measured by self-report, and the shared method variance between MFIS and PSQI may inflate their joint apparent contribution relative to objectively measured grip strength. Fairness disparities are based on subgroups as small as 22 and are not adjusted for multiplicity. Only one participant had PCFS grade 4, so the highest cumulative cut-point of the ordinal model is not interpretable. An inherent limitation of this retrospective cohort is that dates of acute diagnosis and subsequent functional assessment were not captured in the primary records, precluding exact calculation of the elapsed time since infection. Inclusion required persistent symptoms beyond twelve weeks without an upper cutoff, meaning intervals may have varied across participants without the possibility of quantitative adjustment. Consequently, whether the identified correlates, including the inverse hospitalization pattern (Section 4.3), remain stable across different phases of post-COVID recovery or reflect length-biased sampling in outpatient care-seeking remains to be determined in prospective cohorts designed with explicit temporal tracking.

### 4.7. Implications and Future Directions

For research, our findings prioritize fatigue and sleep quality over peak grip strength as candidate targets for prospective post-COVID functional studies. A confirmatory study of an eight-parameter model of this kind would require approximately 566 participants by the criteria applied here (Table 2); investigators planning such work can use this figure directly. Cohorts enrolling at the point of acute infection, rather than at the point of post-COVID consultation, would help resolve the hospitalization association described in Section 4.3, and multidimensional grip protocols capturing fatigability and recovery would test whether dynamic rather than static muscle measures restore the predictive contribution that peak force lacked here [8,39]. Future cohorts should systematically record exact dates of infection and clinical assessment as standard variables, enabling direct evaluation of elapsed time as a covariate or effect modifier to better contextualize acute-severity findings such as hospitalization status (Section 4.6).

For practice, the negative predictive value of 0.500 and the individual-level instability documented in Section 3.6 mean this model is not ready for clinical use, and we make no recommendation to that effect. What the analysis does support is a narrower and, we think, more useful claim for services with constrained resources: systematic assessment of fatigue and sleep quality with brief validated instruments captures more information relevant to functional limitation than dynamometry alone and requires no additional equipment beyond the questionnaires already validated for this population.

### 4.8. Construct Boundaries Between Predictors and Functional Outcome

Interpretation of predictor contributions requires examining construct overlap between self-report inventories and functional grading. Because the MFIS captures functional disruption attributable to fatigue across physical and cognitive domains, its items partially intersect with the daily activity limitations evaluated by the PCFS scale. Accordingly, the strong association observed for the MFIS (Section 3.5, Table 5) represents both shared functional construct variance and direct symptom burden, distinguishing this multidimensional self-report scale from objective physiological markers such as handgrip strength. Other predictors, including the PSQI, hospitalization status, Charlson index, and MoCA, remain conceptually independent of the functional outcome definition. Two fundamental consequences derive from this finding. First, the MFIS result should be interpreted as indicating that the construct measured is closely bound up with functional status, rather than taken as evidence that fatigue, considered independently, directly causes functional limitation, as no causal inference is made in this regard. Second, this overlap does not invalidate this study’s primary comparison because that analysis does not depend on a causal interpretation of the association between the MFIS and general functional status. The central question was whether handgrip strength retained predictive capacity when fatigue and sleep quality were evaluated jointly, and the answer obtained, namely that it provides no additional discrimination, remains unaltered regardless of how the fatigue–function relationship is conceived. Indeed, if construct overlap was to inflate the apparent contribution of the MFIS, this would further weaken, rather than strengthen, the case for handgrip strength as an independent marker. Empirical separation of these constructs was precluded because the predefined eight-parameter methodological budget did not include a tool free from such overlap, and no alternative instrument was administered in this cohort, a limitation that is incorporated into the discussion section as a design recommendation for future studies.

## 5. Conclusions

In this cohort of 130 Colombian adults with post-COVID condition, fatigue emerged as the most influential correlate of functional limitation, with sleep quality and acute-phase hospitalization status also contributing, whereas peak handgrip strength, the variable that motivated the original hypothesis, ranked fifth of eight pre-specified predictors. A parsimonious penalized regression model achieved moderate optimism-corrected discrimination (AUC 0.748, 95% CI 0.669–0.830), acceptable calibration, and positive net benefit across a clinically plausible threshold range; individual-level predictions remained unstable for approximately 30% of participants, and the cohort met 23.0% of the sample size required for confirmatory model development. Reported together with a formal sample size assessment, optimism-corrected internal validation, and joint calibration, utility, stability and fairness analyses, a combination uncommon in post-COVID prediction studies of this size, these hypothesis-generating findings redirect priority toward multidimensional symptom assessment, particularly of fatigue and sleep, ahead of peak strength measurement in future studies of post-COVID functional status, pending confirmation in adequately powered, externally validated cohorts.

## Figures and Tables

**Table 1:** Characteristics of the study population stratified by post-COVID functional status (n = 130).

Variable	Total (n = 130)	No Limitation (n = 39)	Any Limitation (n = 91)	*p* Value
Maximum grip strength (kg)	24.8 [19.3–33.8]	26.0 [21.3–35.4]	23.8 [17.8–30.2]	0.056
Age (years)	52.5 [35.2–63.0]	53.0 [31.0–64.5]	52.0 [38.0–63.0]	0.905
Modified Fatigue Impact Scale (total)	24.0 [9.0–37.0]	9.0 [6.0–25.0]	26.0 [16.0–40.5]	<0.001
Pittsburgh Sleep Quality Index (global)	6.0 [3.0–10.0]	4.0 [2.0–7.5]	7.0 [4.0–12.0]	<0.001
Montreal Cognitive Assessment (total)	25.0 [22.0–27.0]	25.0 [23.0–27.0]	25.0 [22.0–26.5]	0.261
Charlson comorbidity index	0.0 [0.0–3.0]	0.0 [0.0–0.0]	0.0 [0.0–3.0]	0.137
Sex, female	40 (30.8%)	16 (41.0%)	24 (26.4%)	0.147
Sex, male	90 (69.2%)	23 (59.0%)	67 (73.6%)	0.147
Not hospitalized during acute infection	85 (65.4%)	18 (46.2%)	67 (73.6%)	0.005
Hospitalized during acute infection	45 (34.6%)	21 (53.8%)	24 (26.4%)	0.005
Socioeconomic stratum 1	2 (1.5%)	1 (2.6%)	1 (1.1%)	0.756
Socioeconomic stratum 2	57 (43.8%)	19 (48.7%)	38 (41.8%)	0.756
Socioeconomic stratum 3	56 (43.1%)	16 (41.0%)	40 (44.0%)	0.756
Socioeconomic stratum 4	13 (10.0%)	3 (7.7%)	10 (11.0%)	0.756
Socioeconomic stratum 5	2 (1.5%)	0 (0.0%)	2 (2.2%)	0.756
Health insurance: subsidized scheme	93 (71.5%)	28 (71.8%)	65 (71.4%)	1.000
Health insurance: contributory scheme	37 (28.5%)	11 (28.2%)	26 (28.6%)	1.000
Education: primary	22 (16.9%)	6 (15.4%)	16 (17.6%)	0.970
Education: university	30 (23.1%)	10 (25.6%)	20 (22.0%)	0.970
Education: secondary	44 (33.8%)	13 (33.3%)	31 (34.1%)	0.970
Education: technical	34 (26.2%)	10 (25.6%)	24 (26.4%)	0.970
Ethnicity: white	35 (26.9%)	13 (33.3%)	22 (24.2%)	0.713
Ethnicity: Indigenous	6 (4.6%)	2 (5.1%)	4 (4.4%)	0.713
Ethnicity: mixed ancestry	86 (66.2%)	23 (59.0%)	63 (69.2%)	0.713
Ethnicity: Afro-descendant	2 (1.5%)	1 (2.6%)	1 (1.1%)	0.713
Ethnicity: other	1 (0.8%)	0 (0.0%)	1 (1.1%)	0.713

Continuous variables are presented as median [interquartile range]; categorical variables are presented as n (%). *p* values derive from the Mann–Whitney U test (continuous) or the chi-squared test (categorical) and are descriptive only: predictors were pre-specified, and no variable was selected on the basis of a *p* value. Socioeconomic stratum is the Colombian administrative classification of dwellings from 1 (lowest) to 6 (highest); no participant belonged to stratum 6. The subsidized insurance scheme is state-financed for low-income households, and the contributory scheme is financed by payroll contributions. Ethnicity was self-identified: “mixed ancestry” corresponds to mixed Indigenous and European descent, and “Afro-descendant” corresponds to mixed African and European descent, following the categories used in Colombian national statistics. Source: own elaboration.

**Table 2:** Minimum sample size required for model development according to the criteria of Riley et al. by number of candidate parameters.

Candidate Parameters	Criterion 1 (Shrinkage)	Criterion 2 (Optimism)	Criterion 3 (Risk Precision)	Minimum n Required	Minimum Events Required	% of Requirement Met
97	6855	2533	323	6855	2057	1.9%
30	2120	784	323	2120	636	6.1%
15	1060	392	323	1060	318	12.3%
8	566	209	323	566	170	23.0%

Calculations assume an outcome proportion of 0.70 and an anticipated C statistic of 0.715, corresponding to an anticipated Cox–Snell R^2^ of 0.119 (maximum 0.705). The minimum required n is the maximum of the three criteria. The 97-parameter row corresponds to the candidate set considered in an earlier version of this work; the 8-parameter row corresponds to the model reported here. Source: own elaboration.

**Table 3:** Head-to-head comparison of candidate algorithms on identical out-of-fold predictions at the pre-specified decision threshold of 0.70.

Model	AUC (95% CI)	Brier (95% CI)	Cal. Slope/Intercept	Sens./Spec.	PPV/NPV	F1	Bal. Acc.
Ridge logistic (primary)	0.728 (0.625–0.823)	0.183 (0.148–0.220)	0.91/0.00	0.69/0.72	0.85/0.50	0.764	0.705
Lasso logistic	0.723 (0.626–0.819)	0.187 (0.153–0.224)	0.80/0.11	0.65/0.69	0.83/0.46	0.728	0.670
Random forest	0.718 (0.619–0.811)	0.186 (0.152–0.221)	0.95/−0.00	0.68/0.67	0.83/0.47	0.747	0.674
Gradient boosting	0.719 (0.615–0.816)	0.194 (0.151–0.243)	0.49/−0.10	0.75/0.59	0.81/0.50	0.777	0.668
No-skill reference	0.500	0.210	—	1.00/0.00	0.70/—	0.824	0.500

All metrics are computed on out-of-fold predictions from ten-fold cross-validation, so every model is evaluated on the same participants, and none is scored on data it was fitted to. Confidence intervals derive from 1000 bootstrap resamples of participants. The no-skill reference assigns every participant the cohort outcome prevalence. Note that a no-skill model attains F1 = 0.824 and PPV = 0.700 at this prevalence, exceeding all real models; these two metrics should not be interpreted without that comparator. Calibration slope and intercept are reported as slope/intercept, with ideal values 1 and 0 respectively. Abbreviations: AUC, area under the receiver operating characteristic curve; Bal. acc., balanced accuracy; Cal., calibration; CI, confidence interval; NPV, negative predictive value; PPV, positive predictive value; Sens., sensitivity; Spec., specificity. Source: own elaboration.

**Table 4:** Bootstrap internal validation of the primary ridge-penalized logistic model (B = 1000 replicates).

Metric	Apparent	Optimism	Optimism-Corrected	95% CI
AUC	0.794	0.046	0.748	0.669–0.830
Brier score	0.164	−0.021	0.185	0.149–0.230
Calibration slope	1.427	0.277	1.150	0.715–1.694
Calibration intercept	−0.000	0.033	−0.034	−0.498–0.399

The complete modeling pipeline, including multiple imputation, standardization and penalty tuning, was re-executed within each bootstrap replicate. Optimism is the mean difference between apparent performance in the bootstrap sample and performance of the same model in the original cohort. Ideal values are 1 for the calibration slope and 0 for the calibration intercept; both intervals include their ideal value. Source: own elaboration.

**Table 5:** Standardized coefficients and odds ratios from the primary ridge-penalized logistic model.

Predictor	Coefficient (per SD)	Odds Ratio (per SD)
Modified Fatigue Impact Scale (total)	0.483	1.62
Pittsburgh Sleep Quality Index (global)	0.339	1.40
Charlson comorbidity index	0.245	1.28
Age (years)	0.121	1.13
Montreal Cognitive Assessment (total)	0.070	1.07
Sex	0.022	1.02
Maximum grip strength (kg)	−0.155	0.86
Hospitalization during acute infection	−0.331	0.72

Coefficients are expressed per one standard deviation of each predictor, not per raw unit. Because the model is penalized, coefficients are shrunk toward zero and should not be interpreted as unbiased causal effect estimates. Odds ratios above 1 indicate higher odds of any functional limitation. Source: own elaboration.

## Data Availability

The original contributions presented in this study are included in the article, and further inquiries can be directed to the corresponding authors.

## Supplementary Materials

The following supporting information can be downloaded at: https://www.mdpi.com/article/10.3390/biomedicines14091885/s1.
