# Supplementary material for: Beyond Sarcopenia: An Exploratory Machine Learning Analysis of Fatigue, Sleep Quality and Acute-Phase Severity as Correlates of Post-COVID Functional Status in a Colombian Cohort
Source: Biomedicines. 2026 Aug 24;14(9):1885. doi: 10.3390/biomedicines14091885 (PMC13604082; doi:10.3390/biomedicines14091885)
Supplement: Supplementary file 1 [file biomedicines-14-01885-s001.zip › biomedicines-4511357-supplementary.pdf]

Supplementary Materials

The following supporting information is available: Table S1, pairwise algorithmic parity metrics; Table S2, SHAP importance with bootstrap rank stability; Table S3, secondary proportional-odds model coefficients; Table S4, assessment of the proportional-odds assumption across cumulative cut-points; Table S5, missing data among pre-specified predictors; Table S6, completed TRIPOD+AI checklist; Table S7, completed PROBAST+AI assessment; Table S8, complete PCFS grade distribution; Table S9, model-ablation analysis; Table S10, nested-bootstrap threshold-selection uncertainty; Table S11, hospitalization sensitivity analysis adjusted for ICU admission.

Table S1. Pairwise algorithmic parity metrics across sociodemographic attributes (exploratory).

Attribute	Group A	Group B	DPD	DPD 95% CI	DPD>0.10	EOD	EOD 95% CI	EOD>0.10
Sex	Female	Male	0.281	0.11–0.45	Yes	0.318	0.13–0.53	Yes
SES	Low (1-2)	Mid-high (3+)	0.080	0.004–0.26	No	0.066	0.033–0.39	No
Insurance	Subsidized	Contributory	0.116	0.005–0.31	Yes	0.266	0.070–0.50	Yes
Education	Primary	University	0.103	0.003–0.35	Yes	0.300	0.11–0.62	Yes
Education	Primary	Secondary	0.045	0.004–0.28	No	0.462	0.21–0.75	Yes
Education	Primary	Technical	0.107	0.005–0.38	Yes	0.208	0.031–0.56	Yes
Education	University	Secondary	0.058	0.004–0.28	No	0.162	0.041–0.56	Yes
Education	University	Technical	0.004	0.004–0.29	No	0.100	0.040–0.53	No
Education	Secondary	Technical	0.061	0.005–0.30	No	0.262	0.061–0.64	Yes

Metrics computed on out-of-fold predictions across the full cohort (n = 130) at the pre-specified threshold of 0.70. Bootstrap CIs from 1000 resamples of participants; pairs involving the smallest education subgroups have fewer usable replicates (as low as ~693/1000) because a resampled subgroup occasionally fell below the minimum size of 20. Source: own elaboration.

Table S2. SHAP importance with bootstrap rank stability across 200 resamples.

Predictor	Mean |SHAP|	Point rank	Median bootstrap rank	Rank 95% interval	Ranked first (%)
Modified Fatigue Impact Scale (total)	0.402	1	1	1–5	54.0
Hospitalization during acute infection	0.320	2	3	1–7	26.0
Pittsburgh Sleep Quality Index (global)	0.291	3	3	1–7	16.0
Charlson comorbidity index	0.195	4	4	2–8	2.5
Maximum grip strength (kg)	0.121	5	6	2–8	0.5
Age (years)	0.102	6	6	2–8	1.0
Montreal Cognitive Assessment (total)	0.057	7	7	3–8	0.0
Sex	0.036	8	6	3–8	0.0

Mean absolute SHAP values are computed on out-of-fold predictions. The rank interval is the 2.5th to 97.5th percentile of each predictor’s importance rank across bootstrap resamples; a predictor

whose interval spans most of the range cannot be assigned a definite position. SHAP quantifies contribution within the fitted model and is not evidence of biological or causal relevance. Source: own elaboration.

Table S3. Secondary proportional-odds model on the full five-level PCFS scale: standardized coefficients and odds ratios.

Predictor	Coefficient (per SD)	Odds ratio (per SD)
Modified Fatigue Impact Scale (total)	1.056	2.87
Age (years)	0.503	1.65
Charlson comorbidity index	0.286	1.33
Pittsburgh Sleep Quality Index (global)	0.145	1.16
Montreal Cognitive Assessment (total)	0.101	1.11
Sex	-0.218	0.80
Maximum grip strength (kg)	-0.237	0.79
Hospitalization during acute infection	-0.461	0.63

Odds ratios above 1 indicate higher odds of belonging to a more severe PCFS grade, per one standard deviation of the predictor. The optimism-corrected ordinal concordance index was 0.734 (95% CI 0.679–0.784) with apparent value 0.768 and optimism 0.034; McFadden pseudo-R^2 was 0.155. The ordinal concordance index reduces exactly to the area under the curve for a two-level outcome. Source: own elaboration.

Table S4. Assessment of the proportional-odds assumption: predictor coefficients at each cumulative cut-point.

Predictor	Y > 0	Y > 1	Y > 2	Y > 3 †	Range (Y>0 to Y>2)
Sex	-0.021	-0.532	0.591	-2.165	1.124
Pittsburgh Sleep Quality Index (global)	0.525	0.404	-0.415	-1.086	0.940
Age (years)	0.345	0.744	1.243	0.272	0.897
Montreal Cognitive Assessment (total)	0.272	0.175	-0.393	5.867	0.665
Modified Fatigue Impact Scale (total)	0.765	1.380	1.151	4.487	0.615
Maximum grip strength (kg)	-0.133	-0.156	0.167	-4.722	0.323
Charlson comorbidity index	0.391	0.353	0.243	0.125	0.148
Hospitalization during acute infection	-0.495	-0.476	-0.578	-1.960	0.102

Each column reports the coefficient from a separate binary logistic regression at that cumulative cut-point. Under the proportional-odds assumption, coefficients should be similar across cut-points. † The Y > 3 cut-point separates a single participant (PCFS grade 4) from the remaining 129 and is therefore not estimable in any stable sense; its coefficients are reported for completeness but excluded from the range calculation in the final column. This is a descriptive assessment, not a formal test. Source: own elaboration.

Table S5. Missing data among the eight pre-specified predictors.

Variable	Missing (n)	Missing (%)	Missing within analytic sample (n = 130)
Maximum grip strength (kg)	9	6.5	0
Age (years)	9	6.5	0
Sex	9	6.5	0
MFIS total	9	6.5	0
PSQI global	9	6.5	0
MoCA total	9	6.5	0
Charlson comorbidity index	9	6.5	0
Hospitalization during acute infection	9	6.5	0

Missing values in the source file (n = 139) were confined entirely to nine records lacking both outcome and predictor data, which were excluded. Within the analytic sample of 130 participants, there was no missingness among the pre-specified predictors. Multiple imputation by chained equations was nevertheless retained within the modeling pipeline as a pre-specified component. Source: own elaboration.

Table S6. Completed TRIPOD+AI checklist. Reporting follows the TRIPOD+AI [20] statement.

Item	Description	Where reported
1	Title identifies the study as developing a prediction model, the target population and the outcome	Title; identifies the analysis as exploratory, the Colombian cohort and post-COVID functional status
2	Structured abstract	Abstract (246 words), structured as Background, Methods, Results, Conclusions
3a	Background and rationale	Section 1, paragraphs 1–4
3b	Objectives	Section 1, final paragraph (three stated objectives)
4a	Data source and setting	Section 2.1
4b	Study dates and eligibility	Section 2.1
5a	Participants: eligibility criteria	Section 2.1
5b	Participant flow	Section 2.1 (139 screened, 130 analyzed) and Section 3.1
6a	Outcome definition and measurement	Section 2.2
6b	Rationale for outcome dichotomization	Section 2.2 (correspondence to the referral decision; ordinal secondary analysis retained)
7a	Predictors: definition and measurement	Section 2.3
7b	Predictor pre-specification	Section 2.3; rationale for each predictor documented in the version-controlled configuration file
8	Sample size justification	Section 2.4 and Section 3.2; Table 2; Figure 1D
9	Missing data handling	Section 2.3, Section 2.5 (MICE within pipeline); Table S5
10a	Model type and rationale for pre-specification	Section 2.5

Item	Description	Where reported
10b	Predictor handling, tuning and scoring rule	Section 2.5 (log loss rather than AUC; explicit rationale)
10c	Class imbalance handling	Section 2.5: no correction applied, with justification and references
11	Performance measures	Sections 2.6, 2.7
12	Internal validation method	Section 2.6 (bootstrap optimism correction, B = 1000, full pipeline within each replicate)
13	Fairness assessment	Section 2.7; Section 3.7; Figure 4; Table S1
14	Explainability method and its limits	Section 2.7; Section 3.5 (SHAP on out-of-fold predictions with bootstrap rank intervals)
15	Participant characteristics	Table 1; Section 3.1
16	Model specification	Table 5 (coefficients and odds ratios per standard deviation)
17	Model performance	Table 3, Table 4; Figure 2
18	Model updating or recalibration	Not applicable; no updating performed
19	Clinical utility	Section 3.4; Figure 2C
20	Stability of predictions	Section 3.6; Figure 3C
21	Interpretation in context of existing evidence	Sections 4.1–4.4
22	Limitations	Section 4.6 (nine specific limitations)
23	Implications and future work	Section 4.7
24	Data availability	Data Availability Statement
25	Code availability	Data Availability Statement; public repository with synthetic-data generator
26	Funding	Funding statement
27	Conflicts of interest	Conflicts of Interest statement

Table S7. PROBAST+AI self-assessment. Risk of bias was self-assessed using PROBAST+AI [21] for a model development study without external validation. We report the assessment as reached, including the high risk-of-bias rating in the analysis domain, rather than arguing against it.

Domain	Signaling questions considered	Judgement	Rationale
1. Participants	Appropriate data source; appropriate inclusions and exclusions	Low risk	Consecutive adults attending post-COVID services with confirmed prior infection and symptoms beyond 12 weeks. Exclusions were prespecified and non-outcome-dependent. The nine excluded records contained no data.
2. Predictors	Defined and assessed similarly for all participants; assessed without	Low risk	All predictors measured with standardized instruments at a single assessment. Grip strength measured

Domain	Signaling questions considered	Judgement	Rationale
	knowledge of outcome; available at intended time of use		by protocol. Predictors would be available at the point of the intended triage decision.
3. Outcome	Appropriate definition; determined without knowledge of predictors; consistent across participants	Low risk	PCFS is a validated ordinal instrument applied uniformly. Outcome ascertainment did not depend on predictor values.
4. Analysis	Reasonable number of participants with the event; continuous predictors handled appropriately; all enrolled participants included; missing data handled appropriately; univariable selection avoided; overfitting and optimism accounted for; performance measures appropriate	High risk	The cohort meets 23.0% of the minimum sample size requirement for the eight-parameter model (39 events; approximately 5 events per parameter). This alone determines a high risk-of-bias rating and cannot be mitigated analytically. Mitigations applied: predictors pre-specified rather than selected univariably; penalized estimation; multiple imputation within the pipeline; bootstrap optimism correction over 1000 replicates re-executing the full pipeline; calibration, clinical utility and prediction stability all reported with confidence intervals; no class-imbalance correction applied.
Overall risk of bias	—	High risk	Driven entirely by the analysis domain (sample size relative to requirement). Consistent with the exploratory, hypothesis-generating framing of the manuscript.
Applicability	Do participants, predictors and outcome match the intended question and setting?	Low concern for the stated exploratory question; high concern for any clinical application	Applicable to the question of which correlates merit prioritization in future studies. Not applicable to individual clinical decisions: negative predictive value 0.500, and 30.0% of participants change classification across resamples.

Note. A high risk-of-bias rating in the analysis domain is the expected and correct outcome for a development study at this sample size. We report it because concealing it would misrepresent the work and because the exploratory framing of the manuscript is calibrated to it.

Table S8. Complete grade-by-grade distribution of the Post-COVID Functional Status (PCFS) outcome (n = 130).

PCFS grade	Description	n	%
0	No limitation	39	30.0
1	Negligible limitation	33	25.4
2	Slight limitation	45	34.6
3	Moderate limitation	12	9.2
4	Severe limitation	1	0.8

Table S9. Model-ablation analysis: change in out-of-fold discrimination and calibration after removing each pre-specified predictor.

Predictor removed	Full AUC	Reduced AUC	ΔAUC (full−reduced)	ΔAUC 95% CI	Includes 0	ΔBrier (reduced−full)
Modified Fatigue Impact Scale (total)	0.7275	0.6816	0.046	-0.001 to 0.094	Yes	0.011
Hospitalization during acute infection	0.7275	0.6940	0.035	-0.013 to 0.082	Yes	0.009
Pittsburgh Sleep Quality Index (global)	0.7275	0.7078	0.020	-0.011 to 0.053	Yes	0.005
Charlson comorbidity index	0.7275	0.7230	0.005	-0.022 to 0.031	Yes	0.001
Age (years)	0.7275	0.7289	-0.002	-0.018 to 0.014	Yes	-0.003
Sex	0.7275	0.7289	-0.002	-0.009 to 0.007	Yes	-0.002
Montreal Cognitive Assessment (total)	0.7275	0.7360	-0.008	-0.022 to 0.005	Yes	-0.001
Maximum grip strength (kg)	0.7275	0.7374	-0.010	-0.028 to 0.007	Yes	-0.002

Table S10. Nested-bootstrap uncertainty around the 0.70 operating decision threshold (1000 replicates).

Quantity	Value
Originally reported threshold	0.70
Bootstrap-resampled threshold, median	0.71
Bootstrap-resampled threshold, 95% CI	0.66–0.80
Bootstrap-resampled threshold, range	0.54–0.84
Mean net-benefit optimism from threshold selection	0.036
Selection-corrected net benefit at p_t = 0.70, median	0.254
Selection-corrected net benefit at p_t = 0.70, 95% CI	0.152–0.287
Apparent (originally reported) max. incremental net benefit	0.287
Bootstrap replicates completed / requested	1000 / 1000

Table S11. Sensitivity analysis: primary model with ICU admission added as a ninth (exploratory) covariate (n = 130).

Predictor	Coefficient (per SD)	Odds ratio (per SD)
Modified Fatigue Impact Scale (total)	0.478	1.613
Pittsburgh Sleep Quality Index (global)	0.335	1.397
Charlson comorbidity index	0.247	1.280
Age (years)	0.115	1.122
Montreal Cognitive Assessment (total)	0.078	1.081
ICU admission (acute infection)	0.071	1.074
Sex	0.027	1.027
Maximum grip strength (kg)	-0.150	0.861
Hospitalization during acute infection	-0.344	0.709
